# Glomerulus-Selective Regulation of a Critical Period for Interneuron Plasticity in the *Drosophila* Antennal Lobe

**DOI:** 10.1523/JNEUROSCI.2192-19.2020

**Published:** 2020-07-15

**Authors:** Ankita Chodankar, Madhumala K. Sadanandappa, Krishnaswamy VijayRaghavan, Mani Ramaswami

**Affiliations:** ^1^National Centre for Biological Sciences, Tata Institute of Fundamental Research, Bangalore 560065, India; ^2^Trinity College Institute of Neuroscience, School of Genetics and Microbiology, Smurfit Institute of Genetics and School of Natural Sciences, Trinity College Dublin, Dublin 2, Ireland; ^3^Department of Molecular and Systems Biology, Geisel School of Medicine at Dartmouth, Hanover, New Hampshire 03755

**Keywords:** antennal lobe, critical period, long-term habituation, memory, olfaction, plasticity

## Abstract

Several features of the adult nervous systems develop in a “critical period” (CP), during which high levels of plasticity allow neural circuits to be tuned for optimal performance. Through an analysis of long-term olfactory habituation (LTH) in female *Drosophila*, we provide new insight into mechanisms by which CPs are regulated *in vivo*. LTH manifests as a persistently reduced behavioral response to an odorant encountered for 4 continuous days and occurs together with the growth of specific, odorant-responsive glomeruli in the antennal lobe. We show that the CP for behavioral and structural plasticity induced by ethyl butyrate (EB) or carbon dioxide (CO_2_) closes within 48 h after eclosion. The elaboration of excitatory projection neuron (PN) processes likely contribute to glomerular volume increases, as follows: both occur together and similarly require cAMP signaling in the antennal lobe inhibitory local interneurons. Further, the CP for structural plasticity could be extended beyond 48 h if EB- or CO_2_-responsive olfactory sensory neurons (OSNs) are silenced after eclosion; thus, OSN activity is required for closing the CP. Strikingly, silencing of glomerulus-selective OSNs extends the CP for structural plasticity only in respective target glomeruli. This indicates the existence of a local, short-range mechanism for regulating CP closure. Such a local mechanism for CP regulation can explain why plasticity induced by the odorant geranyl acetate (which is attractive) shows no CP although it involves the same core plasticity mechanisms as CO_2_ and EB. Local control of closure mechanisms during the critical period can potentially impart evolutionarily adaptive, odorant-specific features to behavioral plasticity.

**SIGNIFICANCE STATEMENT** The critical period for plasticity represents a stage of life at which animals learn specific tasks or features with particular facility. This work provides fresh evidence that mechanisms for regulating critical periods are broadly conserved across evolution. Thus, a critical period for long-term olfactory habituation in *Drosophila*, which closes early in adulthood can, like the critical period for ocular dominance plasticity in mammals, be extended by blocking sensory neurons early in life. Further observations show that critical periods for plasticity can be regulated by spatially restricted mechanisms, potentially allowing varied critical periods for plasticity to stimuli of different ethological relevance.

## Introduction

During development, brain regions often show increased capacity for plasticity in a temporal window termed the critical period (CP), during which key elements of circuit connectivity are established (Hensch and Fagiolini, 2004). Various forms of learning, such as language learning in humans (Kuhl, 2004) or coordination of sensory inputs in owls (Brainard and Knudsen, 1998), are easier at a young age. Understanding how CPs are regulated, what molecules are involved in closing them, and what core mechanisms govern plasticity in juvenile brains would not only provide a new understanding of developmental neuroscience but also potentially suggest approaches to extend CPs for several forms of learning.

The best studied example of a CP is the ocular dominance plasticity observed following monocular deprivation, which results in visual cortex neurons receiving increased innervation from the open eye (Wiesel and Hubel, 1963). Ocular dominance plasticity occurs most efficiently within a CP of 21–32 d after birth in mice (Gordon and Stryker, 1996) and 15–45 d in rats (Fagiolini et al., 1994). But, rats reared in the dark until postnatal day 60 show ocular dominance plasticity similar to that of normally reared 19- to 21-d-old animals (Fagiolini et al., 1994; Murase et al., 2017), indicating an activity-dependent mechanism for CP closure.

However, much remains to be understood about how plasticity is regulated *in vivo*. In contrast to plasticity following monocular deprivation, the reinnervation of cortical areas receiving sensory input from small, experimentally lesioned regions of the retina by neurons innervating surrounding areas, does not show a CP (Keck et al., 2008). Thus, it remains unclear how local mechanisms involved in CP closure can act in the primary visual cortex as well as how widely CPs can vary within and across sensory and functional modalities. We used the well characterized nervous system of *Drosophila melanogaster* to study CP plasticity with near single-cell resolution.

The ease of genetic manipulation in a specific subset of functionally and anatomically defined neurons in the *Drosophila* olfactory circuit allowed us to gain a unique understanding of local mechanisms involved in CP regulation.

## Materials and Methods

### 

#### 

##### *Drosophila* stocks.

All *Drosophila* cultures were grown on standard cornmeal agar, at 25°C with a 12 h light/dark cycle, and Canton S (CS) flies were used as wild-type controls for all the experiments unless otherwise stated. *GH146-Gal4* (II) was from Reinhard Stocker (University of Fribourg, Fribourg, Switzerland); *Or83b-Gal4* (III), *Gr21a-Gal4* (III), and *VPN-Gal4* (X) were from Leslie Vosshall (Rockefeller University, New York, NY); *UAS-D*α*7-GFP/MKRS* (III) was from Stephen Sigrist (Freie Universität, Berlin, Germany); *LN1-Gal4* (II) was from Kei Ito (Janelia Research Campus, Howard Hughes Medical Institute, Ashburn, VA); *rut^2080^* (X) and *rut^2080^;UAS-rut*^+^(II) flies were from Martin Heisenberg (University of Würzburg, Würzburg, Germany); *UAS-rut RNAi* (VDRC5569) was from the Vienna *Drosophila* Stock Center; *UAS-Kir2.1; TubGal80^ts^* (Chiang et al., 2009); and *Or85a-Gal4* (BDSC23133), *Or82a-Gal4; TM2/TM6Tb* (BDSC23125), *Or42a-Gal4* (BDSC9969), *Pin/CyO; UAS-mCD8::GFP* (BDSC5130), *GH146-QF,QUAS-mCD8::GFP* (III; BDSC30038) were obtained from the Bloomington *Drosophila* Stock Center.

##### Induction of long-term olfactory plasticity.

**Figure 1.**
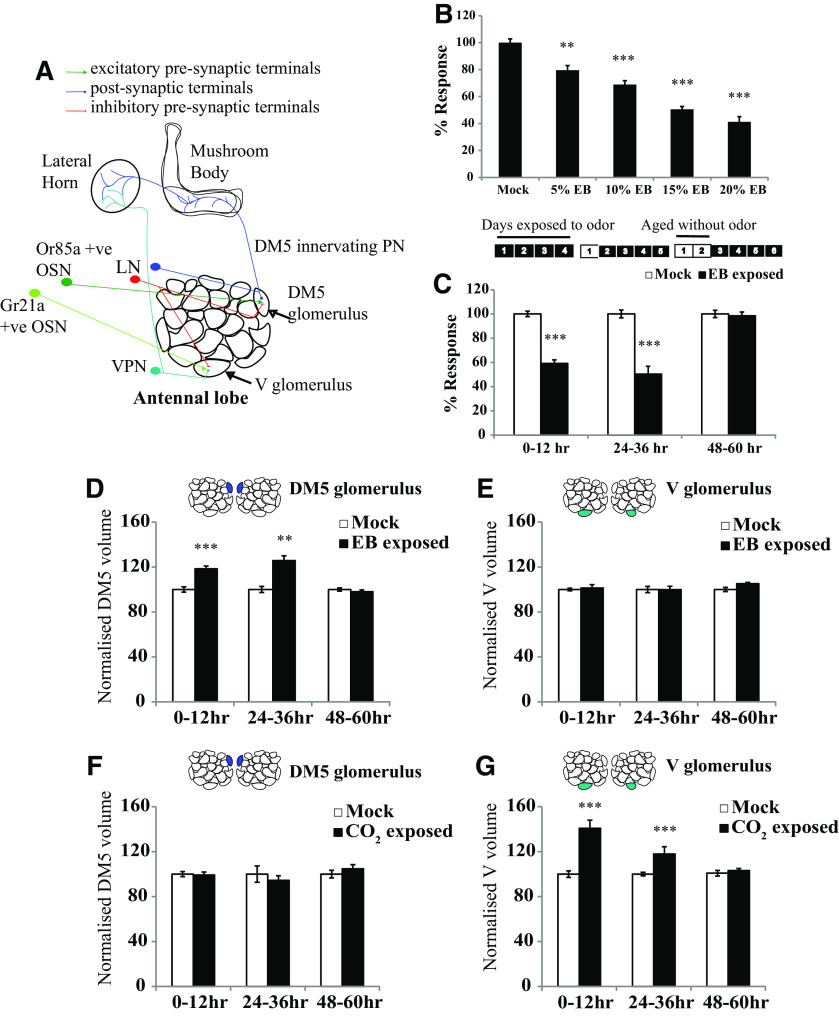
Critical period for LTH in *Drosophila* is closed at 2 d. ***A***, Schematic of the antennal lobe showing OSNs, PNs, and LNs innervating the DM5 and V glomeruli. ***B***, Early optimization of EB exposure conditions to induce long-term habituation. The histogram shows the normalized response of CS flies exposed to EB at the indicated concentrations for 4 d, with odor exposure starting 0–12 h after eclosion. ***C***, Schematics show the odor exposure protocol. The numbers indicate the number of days after eclosion. Shaded boxes indicate that flies were exposed to odor, and unshaded boxes indicate that flies were aged without odor exposure. Normalized response index for LTH behavior of wild-type CS flies that were exposed to EB at different time periods after eclosion such as 0–12, 24–36, and 48–60 h after eclosion. *N* is 8–11 sets for each bar. Refer to Table 2 for actual response index (RI) values, *p* values, and *n* values. ***D–G***, Histogram shows the quantification of glomerular volume in mock and odor-exposed flies. The shaded glomeruli in the antennal lobe schematic indicate the glomerulus analyzed after odor exposure. Normalized volume for the EB-sensitive DM5 glomerulus and CO_2_-sensitive V glomerulus measured after 4 d of EB (***D***, ***E***) and CO_2_ (***F***, ***G***) exposure. Refer to Table 3 for normalized volume values, *p* values, and *n* values. ***p ≤ 0.001, **p ≤ 0.01, and *p ≤ 0.05 determined by Student's *t* test. Error bars indicate the mean ± SEM. *N* is 7–27 glomeruli for each bar.

We used previously described odorant exposure protocols with minor modifications (Das et al., 2011; Kidd et al., 2015). Briefly, to establish the CP for memory acquisition, flies were exposed to odorants [ethyl butyrate (EB) or carbon dioxide (CO_2_)] for 4 d starting from 0–12, 24–36, or 48–60 h after eclosion. Flies were aged at 25°C for the appropriate duration before odorant exposure. Ethyl butyrate (catalog #101343650, Sigma-Aldrich) exposure was conducted by suspending a 1.5 ml centrifuge tube containing 1 ml of 20% of EB (diluted in paraffin oil) covered with perforated cling film in a bottle containing standard *Drosophila* medium with the flies to be tested, which was sealed with a tight-fitting cotton plug. Control “mock-exposed” flies were identically treated, but exposed to 1 ml of paraffin oil (solvent) in the tube. CO_2_ exposure was conducted in a tissue culture incubator, maintained at 5% CO_2_. Mock-treated flies were exposed to air. After 4 d of odor exposure, the flies were then starved overnight in empty vials containing a moist filter paper, before being tested for their olfactory responses or glomerular volumes. Previously established odor–choice behavioral assays for measuring the olfactory responses and long-term olfactory habituation (LTH) phenotypes were used for testing the CP (Das et al., 2011). In choosing the protocols above, we not only chose conditions that allow our observations to be compared with prior publications but also built on prior work optimizing protocols for induced long-term plasticity for each odorant. Prior work has shown that LTH is best induced by 4 d exposure to 20% EB or 5% CO_2_, but only unreliably at concentrations <5% EB or 2.5% CO_2_ (Das et al., 2011; Fig. 1*B*). Photo-ionization detector measurements (Table 1)indicate that concentrations for EB in the odorant chamber with “20% EB” range from 1231 ppm on the first day after odor exposure to 727 ppm on the fourth day after odor exposure. CO_2_ concentrations are absolute and constant because exposure is in a CO_2_ incubator. Geranyl acetate (GA) concentrations are as previously defined and used in the study by Kidd et al. (2015).

**Table 1.** Concentration of EB in odor exposure chamber

Day of odor exposure	Concentration of EB
Day 1	1231 ppm
Day 2	1231 ppm
Day 3	995 ppm
Day 4	727 ppm

Direct measurements of odorant concentration as a function of time during odor exposure. The table shows EB concentration in odor exposure chambers on each day of odor exposure, determined using photoionization diode measurements.

For studying the CP extension, flies carrying *UAS-Kir2.1; Tub-Gal80^ts^* transgenes raised at 18°C were collected within 12 h of eclosion and were kept at either 22°C (temperature control) or 29°C (experimental) for 48 h, as indicated in the figures. These 48- to 60-h-old flies were then exposed to odors at room temperature (RT), as described above.

##### Immunohistochemistry and confocal imaging.

Fly brains were dissected in 1× PBS and fixed in 4% EM grade paraformaldehyde prepared in 1× PBS with 0.3% Triton-X (PTX) for 30 min at RT. Later, the samples were washed in 0.3% PTX for 1 h at RT and incubated with primary antibodies for 2 d on a shaker at 4°C. The primary antibodies were removed and four 0.3% PTX washes of 15 min duration were given, before adding secondary antibody. Samples were incubated with fluor-conjugated secondary antibodies overnight at 4°C on a shaker. The samples were then washed with 0.3% PTX for 1 h at RT before mounting in Vectashield (H-1000, Vector Laboratories) on slides with spacers. The 512 × 512 with 0.5 µm interval images were acquired using a 60× objective on Olympus FV1000 Confocal Microscope or a 63× objective on a Zeiss LSM 510 Meta Microscope.

The following primary antibodies were used: Invitrogen rabbit anti-GFP (1:10,000; Thermo Fisher Scientific; or 1:800; Abcam); rabbit anti-dsRed (1:800; Clontech); and mouse anti-bruchpilot (1:20; mAbnc82, Developmental Studies Hybridoma Bank). The following secondary antibodies were used: Invitrogen goat anti-rabbit Alexa Fluor 488 (Thermo Fisher Scientific; RRID:AB_2534122); and Invitrogen goat anti-mouse Alexa Fluor 647 (Thermo Fisher Scientific; RRID:AB_2535804), both used at 1:400 dilution.

##### Glomerular volume measurements.

Glomerulus volume measurement was performed as previously described (Das et al., 2011). Dissected brains from mock and odor-exposed flies were imaged using an Olympus FV1000 Confocal Microscope with the same acquisition settings. The images were imported into the image analysis software Amira version 5.2.0 (Thermo Fisher Scientific). Glomeruli were identified based on anti-BRP staining and labeled on the *x–y*, *x–z*, and *y–z* axes. The glomeruli were then 3D reconstructed, and the total volume was calculated. We used a yoked experimental design with internal controls to estimate glomerular growth. Thus, the volumes for identically and synchronously handled mock and odor-exposed flies were normalized to mean volume for the mock-treated flies and expressed as a percentage volume. All volume measurements were performed blinded for experimental conditions. Female flies were dissected for all experiments, except for *rut^2080^* mutants, where males were dissected. More than 10 glomeruli were analyzed for all experiments unless otherwise indicated. An unpaired Student's *t* test was used to compare the glomerular volumes of mock and odor-exposed flies. The *p* values are indicated as *p ≤ 0.05, **p ≤ 0.01, and ***p ≤ 0.001, and as ns for nonsignificance (*p* > 0.05).

##### Fluorescence quantification.

Confocal images to quantify the fluorescence were acquired for mock and odor-exposed flies for each genotype using the same imaging parameters. All experiments were performed as described in the section for glomerular volume measurement. The confocal stacks were imported to FIJI, and a custom program (available at https://gitlab.com/umesh-NCBS/pixel_value_getter) was used to label the glomerulus of interest in the channel showing anti-BRP staining. The corresponding pixels in the GFP channel were selected and the number of pixels with intensity values above background levels within the ROIs was counted for each glomerulus. ROIs in neuropil areas other than the antennal lobes were selected as background for each confocal stack. An unpaired Student's *t* test was used to compare mock and odor-exposed flies with 0.05 as the statistical significance level.

## Results

### Critical periods for long-term olfactory habituation to ethyl butyrate and CO_2_ close 2 d after eclosion

Drosophila detect odorants largely via ∼2000 olfactory sensory neurons (OSNs) present on the antenna and maxillary palp, which may be further subdivided into ∼60 OSN types based on the odorant receptor expressed. Each OSN type projects to a distinct “glomerulus” in the insect antennal lobe, where it makes synapses onto uniglomerular projection neurons (PNs) that in turn connect to the deeper brain centers, such as mushroom body and lateral horn. OSNs and PNs activate predominantly multiglomerular local interneurons, of which LN1 and LN2 define two subtypes that provide feedforward or feedback inhibition to the antennal lobe (Das et al., 2011; Fig. 1*A*). A 4 d exposure to the aversive odorants benzaldehyde, isoamyl acetate, CO_2_, or EB results in a long-lasting decrement in behavioral response to respective odorants, referred to as LTH. This reduced behavioral response in adult *Drosophila* occurs in association with odorant-responsive glomerular volume changes (Devaud et al., 2001, 2003; Sachse et al., 2007; Das et al., 2011). Previous studies have established that there is often a CP in early adulthood during which *Drosophila* are capable of exhibiting olfactory LTH. For example, long-term behavioral and structural plasticity are observed if 4 d benzaldehyde or CO_2_ exposure is started on the first day after eclosion, but not if odor exposure is started 8 d after eclosion (Devaud et al., 2003; Sachse et al., 2007). However, the exact temporal window for this critical period plasticity has not been clearly defined (Devaud et al., 2001, 2003; Sachse et al., 2007; Das et al., 2011).

**Table 2.** Response index of CS flies exposed to EB at specified time points

4 d EB exposure started at	Raw RI values		*p* value
	Mock (*n*)	EB exposed (*n*)	
0–12 h	0.69 ± 0.01 (10)	0.41 ± 0.02 (11)	*p* < 0.001
24–36 h	0.65 ± 0.02 (9)	0.33 ± 0.04 (10)	*p* < 0.001
48–60 h	0.69 ± 0.02 (8)	0.68 ± 0.02 (11)	*p* = 0.727

Raw values for response index (RI) of CS flies exposed to 20% EB for 4 d starting at 0–12, 24–36, and 48–60 h after eclosion.

**Table 3.** Normalized glomerular volumes

Genotype	Temperature	Exposure started from	Exposed to	Glomeruli measured	Mock (*n*)	Exposed (*N*)	*p* value
*CS*	RT	0–12 h	EB	DM5	100.00 ± 3.13 (25)	119.65 ± 3.67 (27)	2.82E-06
*CS*	RT	0–12 h	EB	DM2	100.00 ± 2.41 (17)	105.84 ± 1.45 (20)	0.04762
*CS*	RT	0–12 h	EB	VM7	100.00 ± 6.38 (7)	98.91 ± 1.72 (8)	0.8328
*CS*	RT	0–12 h	EB	V	100.00 ± 5.55 (14)	102.93 ± 7.16 (13)	0.7035
*CS*	RT	24–36 h	EB	DM5	100.00 ± 2.69 (10)	125.69 ± 4.24 (7)	0.00037
*CS*	RT	24–36 h	EB	V	100.00 ± 2.79 (6)	99.87 ± 3.02 (7)	0.9760
*CS*	RT	48–60 h	EB	DM5	100.00 ± 1.45 (19)	98.03 ± 1.62 (22)	0.36903
*CS*	RT	48–60 h	EB	V	100.00 ± 1.28 (9)	105.17 ± 0.75 (9)	0.0344
*CS*	RT	0–12 h	CO_2_	V	100.00 ± 2.89 (12)	140.84 ± 7.22 (14)	6.5447E-05
*CS*	RT	0–12 h	CO_2_	DM5	100.00 ± 2.25 (7)	99.14 ± 2.76 (10)	0.8112
*CS*	RT	24–36 h	CO_2_	V	100.00 ± 1.64 (8)	117.99 ± 6.41 (6)	0.0324
*CS*	RT	24–36 h	CO_2_	DM5	100.00 ± 7.28 (7)	94.36 ± 4.13 (7)	0.5118
*CS*	RT	48–60 h	CO_2_	V	100.00 ± 2.46 (12)	103.17 ± 1.79 (12)	0.3094
*CS*	RT	48–60 h	CO_2_	DM5	100.00 ± 4.05 (10)	104.75 ± 3.67 (8)	0.3539
*CS*>*Kir2.1, tubGal80^ts^*	29°C	48–60 h	EB	DM5	100.00 ± 4.38 (11)	103.98 ± 3.73 (14)	0.4966
*CS*>*Kir2.1, tubGal80^ts^*	29°C	48–60 h	EB	V	100.00 ± 4.65 (12)	103.34 ± 3.56 (9)	0.5748
*Or83b*>*Kir2.1, tubGal80^ts^*	RT	48–60 h	EB	DM5	100.00 ± 5.05 (7)	102.2 ± 5.65 (7)	0.7765
*Or83b*>*Kir2.1, tubGal80^ts^*	RT	48–60 h	EB	V	100.00 ± 3.68 (6)	100.99 ± 3.76 (8)	0.8557
*Or83b*>*Kir2.1, tubGal80^ts^*	29°C	48–60 h	EB	DM5	100.00 ± 5.43 (10)	124.36 ± 4.4 (19)	0.0428
*Or83b*>*Kir2.1, tubGal80^ts^*	29°C	48–60 h	EB	V	100.00 ± 3.15 (5)	98.75 ± 0.99 (8)	0.8345
*Or85a*>*Kir2.1, tubGal80^ts^*	RT	48–60 h	EB	DM5	100.00 ± 3.08 (10)	104.73 ± 4.23 (9)	0.3784
*Or85a*>*Kir2.1, tubGal80^ts^*	RT	48–60 h	EB	V	100.00 ± 1.43 (9)	111.11 ± 3.72 (8)	0.0789
*Or85a*>*Kir2.1, tubGal80^ts^*	29°C	48–60 h	EB	DM5	100.00 ± 4.11 (18)	122.74 ± 4.28 (21)	0.0003
*Or85a*>*Kir2.1, tubGal80^ts^*	29°C	48–60 h	EB	DM2	100.00 ± 5.64 (8)	118.2 ± 3.34 (10)	0.5664
*Or85a*>*Kir2.1, tubGal80^ts^*	29°C	48–60 h	EB	DL5	100.00 ± 3.33 (10)	100.51 ± 4.27 (10)	0.9261
*Or85a*>*Kir2.1, tubGal80^ts^*	29°C	48–60 h	EB	V	100.00 ± 2.7 (14)	104.2 ± 2.73 (15)	0.2838
*Or85a*>*Kir2.1, tubGal80^ts^*	29°C	48–60 h	CO_2_	DM5	100.00 ± 3.22 (8)	111.51 ± 4.51 (8)	0.2389
*Or85a*>*Kir2.1, tubGal80^ts^*	29°C	48–60 h	CO_2_	V	100.00 ± 5.96 (7)	114.05 ± 5.98 (6)	0.0588
*Gr21a*>*Kir2.1, tubGal80^ts^*	RT	48–60 h	CO_2_	V	100.00 ± 3.27 (15)	100.35 ± 8.07 (10)	0.9684
*Gr21a*>*Kir2.1, tubGal80^ts^*	RT	48–60 h	CO_2_	DM5	100.00 ± 3.75 (8)	97.77 ± 9.26 (7)	0.8293
*Gr21a*>*Kir2.1, tubGal80^ts^*	29°C	48–60 h	CO_2_	V	100.00 ± 5.13 (14)	144.24 ± 10.61 (16)	0.0011
*Gr21a*>*Kir2.1, tubGal80^ts^*	29°C	48–60 h	CO_2_	DM5	100.00 ± 4.11 (12)	114.94 ± 7.74 (11)	0.4456
*Gr21a*>*Kir2.1, tubGal80^ts^*	29°C	48–60 h	EB	V	100.00 ± 4.6 (6)	97.13 ± 3.46 (6)	0.0586
*Gr21a*>*Kir2.1, tubGal80^ts^*	29°C	48–60 h	EB	DM5	100.00 ± 2.76 (9)	97.88 ± 4.04 (12)	0.2389

Normalized volumes of glomeruli of the indicated genotype after 4 d of odor exposure starting at 0–12, 24–36, and 48–60 h after eclosion. Raw volume values are shown in Extended Data Table 3-1.

10.1523/JNEUROSCI.2192-19.2020.tab3-1 Table 3-1: Glomerular volume values for indicated genotypes and experimental conditions. Volumes of glomeruli (in μm^3^) of the indicated genotype after 4 d of odor exposure starting at 0–12, 24–36, and 48–60 h after eclosion. Download Table 3-1, DOC file.

We, therefore, performed experiments to better define temporal features of CP plasticity to EB and CO_2_ in the well defined antennal lobe circuit, where LTH-associated structural changes occur (Das et al., 2011; Fig. 1*A*). To determine the earliest time point at which the CP closes, we exposed adult flies to 20% EB for 4 d as previously described (Das et al., 2011) starting at the following alternative time points: 0–12 h after eclosion; 24–36 h after eclosion; or 48–60 h after eclosion. The behavioral responses of these flies were then tested at 10^−3^ concentration of EB using the “Y-maze” assay (Das et al., 2011).

Flies exposed to EB at 0–12 and 24–36 h after eclosion showed a significant reduction in the behavioral response to EB compared with mock controls. However, if 4 d of odor exposure were started 48–60 h after eclosion, then the odor responses of EB-exposed flies and controls were indistinguishable (Fig. 1*C*, Table 2). Thus, the CP for LTH to EB closes within 2 d after eclosion.

Since, previous work has shown that the antennal lobe neurons contribute to olfactory habituation (Das et al., 2011), we asked whether structural changes induced by 4 d of odorant exposure also occurred only within the same CP. In animals where odorant exposure was initiated at different ages, we stained the dissected brains with a neuropil-specific antibody (nc82/anti-BRP), visualized them in 3D with confocal microscopy, and measured the volumes of glomeruli known to respond to the repulsive odorants EB and CO_2_. Flies exposed to EB starting 0–12 or 24–36 h after eclosion show an increase in the volume of the EB-responsive DM5 glomerulus (0–12 h: 933.48 ± 29.25 µm^3^ for mock vs 1116.93 ± 34.25 µm^3^ for EB-exposed flies, *p* < 0.001; 24–36 h: 809 ± 21.75 µm^3^ for mock vs 1016.86 ± 34 µm^3^ for EB-exposed flies, *p* < 0.001), but the same flies show no significant changes in volume of the CO_2_-responsive V glomerulus (0–12 h: 3372.57 ± 187.11 µm^3^ for mock vs 3471.31 ± 242.51 µm^3^ for EB-exposed flies, *p* = 0.703; 24–36 h: 2242.83 ± 62.5 µm^3^ for mock vs 2240 ± 67.7 µm^3^ for EB-exposed flies, *p* = 0.976), which does not respond to EB (Fig. 1*D*,*E*, first and second pair of bars, Table 3). Correspondingly, flies exposed to CO_2_ starting at these time points also show glomerular growth, but in the CO_2_-responsive V glomerulus (0–12 h: 2205 ± 63.82 µm^3^ for mock vs 3105.5 ± 159.26 µm^3^ for CO_2_-exposed flies, *p* < 0.001; 24–36 h: 2301 ± 37.85 µm^3^ for mock vs 2715 ± 147.39 µm^3^ for CO_2_-exposed flies, *p* < 0.001), not in the EB-responsive DM5 (0–12 h: 1078.29 ± 24.27 µm^3^ for mock vs 1069 ± 29.79 µm^3^ for CO_2_-exposed flies, *p* = 0.811; 24–36 h: 747.83 ± 54.47 µm^3^ for mock vs 705.67 ± 30.88 µm^3^ for CO_2_-exposed flies, *p* = 0.512; Fig. 1*F*,*G*, first and second pair of bars, Table 3). In contrast, if flies were exposed to either EB or CO_2_ for 4 d, starting 48 h after eclosion, then there was no significant difference in glomerular volumes compared with their mock-exposed controls (Fig. 1*D–G*, third pair of bars, Table 3). All glomerular volumes were normalized for each pair of mock and odor-exposed flies where the average volume of the mock exposed glomeruli was taken as 100%. Normalization of glomerular volume was necessary because flies grown at different times can show variation in glomerular volumes (Sachse et al., 2007).

Together, our data indicate that odor exposure to EB or CO_2_ must begin before flies are ∼48 h old for long-term behavioral habituation and structural plasticity in odorant-responsive (respectively DM5 and V) glomeruli of the antennal lobe. Thus, a CP for long-term plasticity for the repulsive odorants EB and CO_2_ closes ∼48 h (i.e., 2 d) after eclosion.

### Effects of EB exposure on OSN innervation in antennal lobe glomeruli

**Figure 2.**
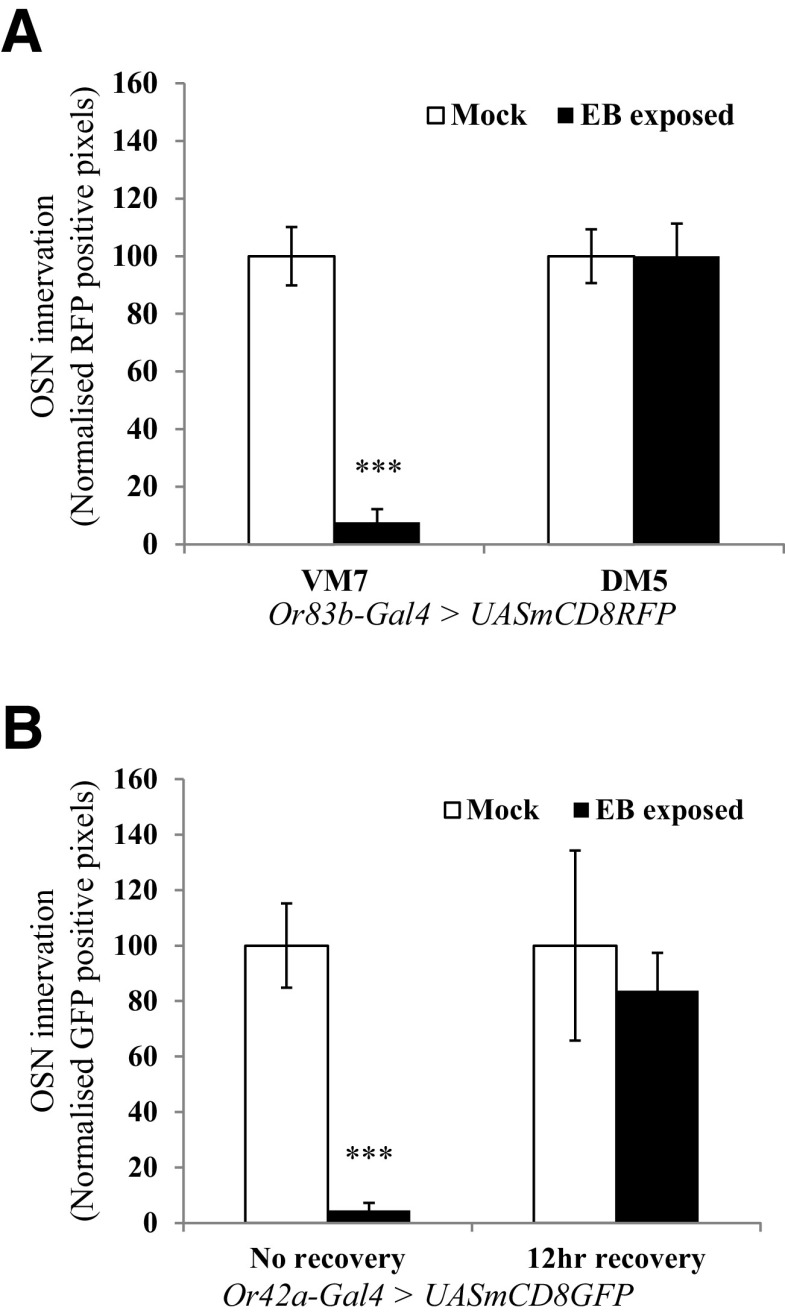
Quantification of OSN innervation in antennal lobe glomeruli. ***A***, OSN innervation after 2 d of EB exposure. OSNs that innervate various olfactory glomeruli were visualized in *Or83b-Gal4*> *UAS-mCD8 RFP* flies, using immunohistochemistry and confocal microscopy. Bar graph shows normalized RFP-positive pixels in VM7 and DM5 glomeruli of the same animals. ***B***, Innervation of Or42a-positive OSNs is rapidly restored within 12 h after recovery after 4 d of odorant exposure. *Or42a-Gal4* > *UAS-mCD8GFP* flies were exposed to EB for 4 d starting 0–12 h after eclosion, and then one group was allowed 12 h of recovery in air before dissection. Bar graphs show the extent of OSN innervation in VM7 glomeruli with and without 12 h of recovery. Error bars indicate the mean ± SEM. *p ≤ 0.05, **p ≤ 0.01, ***p ≤ 0.001, determined by Student's *t* test.

We did not observe any changes in volumes of VM7 glomeruli examined 12–24 h after 4 d exposure to 20% EB (Table 3). Consistent with a recent report (Golovin et al., 2019), we observed a decrease in Or42a OSN innervation and synapses in the VM7 glomerulus immediately after odor exposure, which recovers 12 h after EB exposure (Fig. 2*B*). We also found that flies exposed to EB for 48 h after eclosion show decreased innervation of the VM7 glomerulus, compared with mock-exposed controls, but there is no difference in DM5 innervation in the same flies (Fig. 2*A*).

### Glomerular volume changes occur together with increased PN arborization in odorant-selective glomeruli, which requires *rutabaga* function in GABAergic local interneurons

Many lines of previously published evidence support a model in which increased glomerular volume arises from increased numbers of inhibitory synapses made between the LN1 subclass of local interneurons and PN dendrites through a process that requires cAMP signaling in LN1 (Sachse et al., 2007; Das et al., 2011; Sudhakaran et al., 2012). Such an increase in LN1 synapses is predicted to be accompanied by a parallel increase in PN arborization and postsynaptic contact sites in the glomeruli that show volume increase. To directly address the cellular basis for the observed structural change, we therefore examined whether changes in PN processes and their postsynaptic elements could be observed during glomerular plasticity as well as whether such increases, if observed, required cAMP signaling in LN1 neurons.

**Figure 3.**
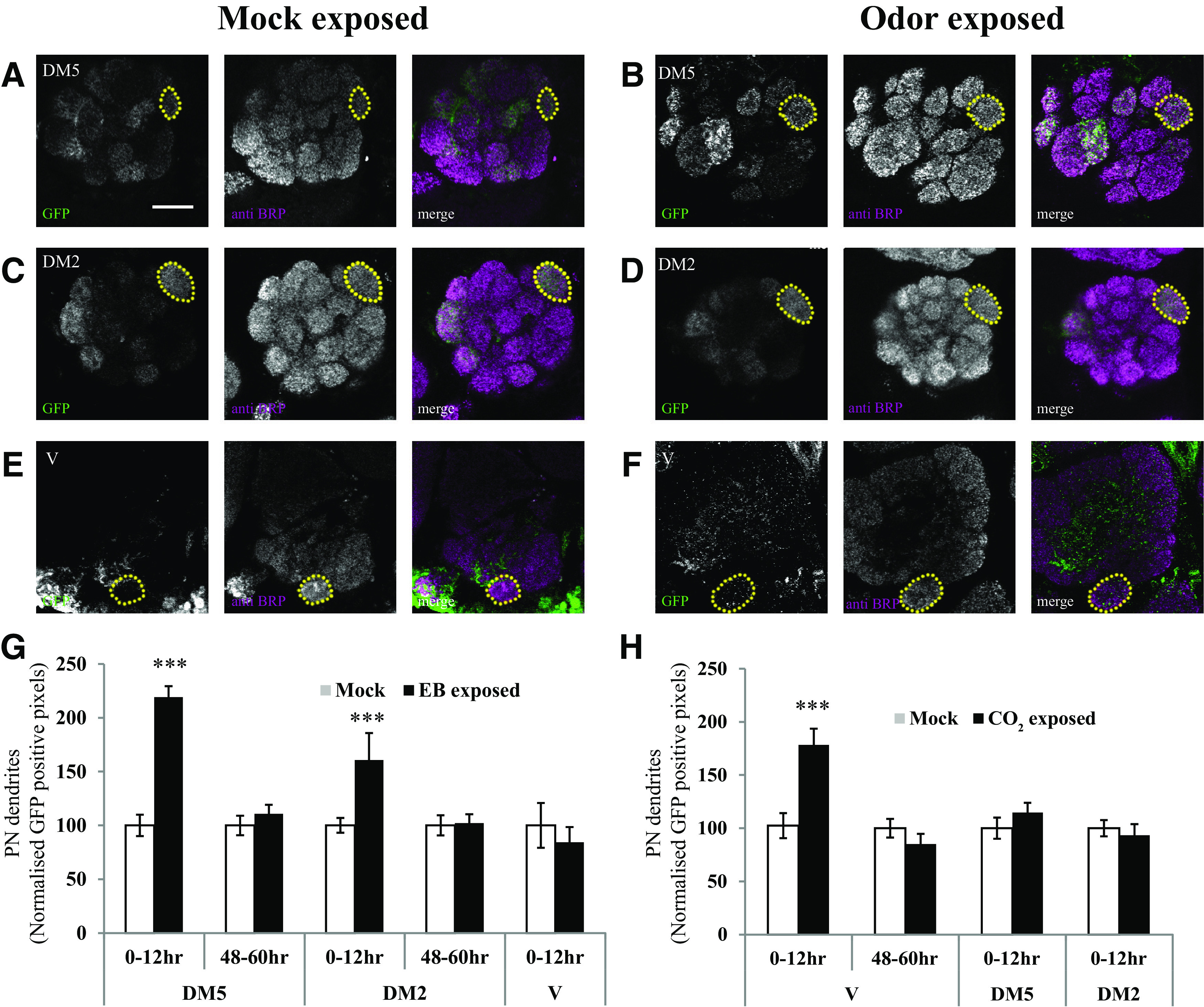
Projection neuron processes are elaborated in odorant-specific glomeruli during the critical period after LTH formation. PN postsynaptic terminals were labeled using an acetylcholine receptor subunit tagged with GFP (Dα7-GFP). ***A–F***, Confocal image showing *GH146-Gal4* > *UAS-D*α*7-GFP* (***A–D***) and *VPN-Gal4* > *UAS-D*α*7-eGFP* (***E***, ***F***) in the antennal lobe. ***A–D***, Yellow dotted lines mark the boundary of EB-responsive, GH146-positive DM5 glomerulus in flies (0–12 h) exposed to mock (***A***) and EB (***B***), and DM2 glomerulus in flies exposed to mock (***C***) and EB (***D***). ***E***, ***F***, CO_2_-selective V glomerulus in flies (0–12 h) exposed to mock (***E***) and CO_2_ (***F***). Scale bar, 20 µm. PN postsynaptic processes showed an increase in DM5, DM2 (***B***, ***D***), and V (***F***) glomeruli after EB and CO_2_ exposure compared with mock-exposed controls, respectively. ***G***, ***H***, Histogram shows GFP-positive pixels in DM5, DM2, and V glomeruli after EB (***G***) and CO_2_ (***H***) exposure at different times after eclosion. The white bar corresponds to mock exposure, whereas the black bar corresponds to odor exposure. *p ≤ 0.05 and ***p ≤ 0.001, determined by Student's *t* test. Error bars indicate the mean ± SEM.

We visualized either PN processes by driving the expression of CD8-GFP, a nonspecific membrane marker, or postsynaptic domains of PN dendrites by driving the expression of Dα7-GFP, a GFP-tagged nonfunctional acetylcholine receptor subunit, in selected subsets of PNs using the *Drosophila* GAL4 system (Fayyazuddin et al., 2006) The GH146-promoter (Stocker et al., 1990) was used to target EB-responsive PNs and the VPN promoter for CO_2_-sensitive PNs (Fig. 3*A–F*). While Dα7-GFP would be preferentially localized to excitatory postsynapses, we expected that, particularly when overexpressed, it could localize to dendrites that also receive GABAergic inputs. Moreover, being predominantly localized to synapses, it could provide a more selective measure of postsynapses than CD8-GFP.

**Figure 4.**
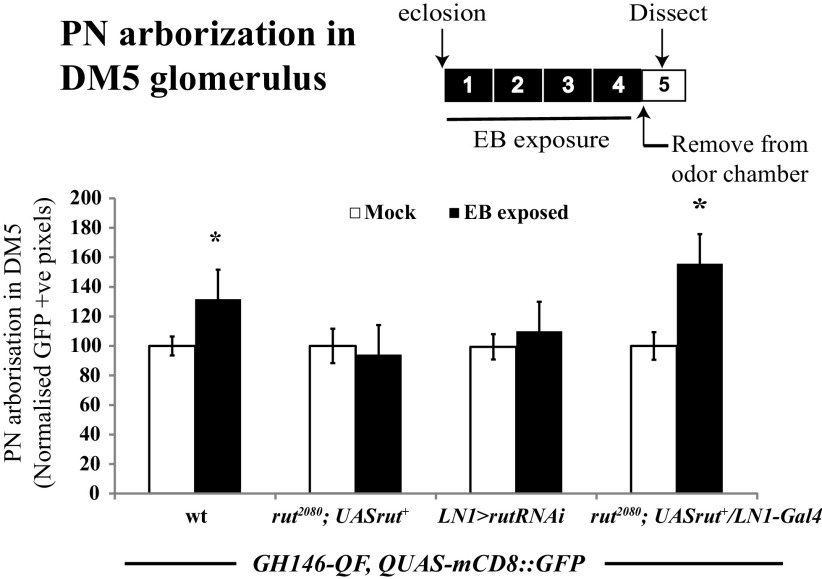
*rutabaga* function is required in GABAergic LNs for the elaboration of PN processes. Bar graphs show GFP-positive pixels in DM5 glomerulus of mock and EB-exposed *GH146QF, QUASmCD8::GFP*/+ flies, without any perturbation, *rut^2080^* mutants, LN1-specific *rutabaga* knockdown, and *rutabaga* mutants expressing *UAS-rut*^+^ transgene in LN1 neurons. The white bar corresponds to mock exposure, whereas the black bar corresponds to EB exposure. Error bars indicate the mean ± SEM. *p ≤ 0.05, determined by Student's *t* test.

Following 4 d of odorant exposure of young flies (0–12 h) to EB or CO_2_, PN processes showed a substantial increase in CD8-GFP or Dα7-GFP fluorescence specifically within odorant-responsive glomeruli (Figs. 3*A–H*, 4). Thus, in flies exposed to EB starting at 0–12 h after eclosion, increased fluorescence could be seen in EB-responsive DM5 (100 ± 10% control vs 219 ± 10% EB-exposed, *p* < 0.001) and DM2 (100 ± 7% control vs 160 ± 25% EB-exposed, *p* < 0.001) glomeruli, but not in V glomerulus (Fig. 3*G*). Similarly, increased fluorescence was observed in the V glomerulus after CO_2_ exposure compared with mock exposed controls (100 ± 12% for mock vs 178 ± 15% for CO_2_-exposed flies, *p* < 0.001; Fig. 3*H*). These increases were not seen in flies exposed to odors starting 48–60 h after eclosion and also no significant differences were observed in the innervation of control glomeruli for either time point (Fig. 3*G*,*H*). Together, these observations indicate that EB- or CO_2-_ induced increases in PN arborization requires odorant exposure within a CP after eclosion that matches the CP for behavior.

Similar, but less dramatic results were also observed when PN processes, rather than postsynapses, were visualized with the membrane marker CD8::GFP (100 ± 6% for mock vs 131 ± 11% for EB-exposed flies, *p* < 0.025; Fig. 4). In contrast, in *rutabaga* mutants (*rut^2080^*), the PN signal in DM5 glomerulus was not significantly different between mock and EB-exposed flies (Fig. 4).

To further confirm that the observed odor-induced PN plasticity reflects processes that underlie glomerular growth, we asked whether the former requires cellular plasticity pathways identical to ones previously implicated in LTH and glomerular growth (Das et al., 2011). In particular, we tested whether *rutabaga* signaling (*rut*), which encodes a Ca^2+^/calmodulin-responsive adenylyl cyclase known to be required in the LN1 subset of inhibitory local neurons for LTH and glomerular growth (Das et al., 2011) was also required in these same neurons for the increased arborization of PN processes. We manipulated *rut* in LN1 neurons using the GAL4/UAS system, while monitoring PN processes using a parallel QF/QUAS system (Potter et al., 2010) in the same flies, to address this question experimentally.

Two lines of data indicate that *rut* is required in LN1 interneurons for odorant-induced PN arborization. First, animalsin which *rut* expression was selectively knocked down in LN1 interneurons using transgenic RNAi (*LN1-Gal4/UAS-rutRNAi; GH146-QF, QUAS-mCD8::GFP*) showed no odor-induced PN growth (Fig. 4). Second, if a *rut*^+^ transgene was expressed specifically in LN1 neurons of *rut^2080^* mutants (*rut^2080^; LN1-Gal4/UAS-rut^+^; GH146-QF, QUAS-mCD8::GFP*), then EB exposure for the first 4 d after eclosion (starting at 0–12 h after eclosion) led to increased PN arborization in the DM5 glomerulus (Fig. 4). Thus, in odorant-exposed flies, expression of the *rutabaga-*encoded adenylate cyclase is necessary and sufficient in LN1 neurons for the odor-induced elaboration of PN processes, exactly as previously observed for EB- and CO_2_-induced LTH and glomerular growth.

Together, two lines of data argue that the glomerular volume increase observed after EB LTH (Das et al., 2011) reflects increased arborization of PN dendrites in the DM5 glomerulus. First, odor-induced glomerular volume changes as well as increased PN arborization show the same critical period and are seen only if odorant exposure is initiated during a CP <48 h after eclosion (Figs. 1, 3). Second, *rutabaga/*adenylate cyclase signaling is required in the LN1 subset of local interneurons both for glomerular growth and increased PN arborization (Figs. 1, 4). The latter observation is particularly informative, because previous work has shown that *rutabaga* requirement in LTH is highly specific for LN1, being dispensable in OSN, PN, or LN2 neurons.

### OSN silencing extends the critical period

Given that dark rearing is well known to extend the duration of the CP for visual plasticity in the V1 area of the mammalian cortex (Cynader, 1983; Fagiolini et al., 1994), we examined whether similar silencing of olfactory inputs in *Drosophila* would extend the CP for odorant-induced plasticity for >48 h. Note that OSN silencing is also expected to silence PN and LN targets because, in *Drosophila* and other insects, OSNs drive most electrical activity in the antennal lobe circuit (Wilson, 2004; Joseph et al., 2012).

We used *Or83b-Gal4* to drive the inward-rectifying potassium channel *UAS-Kir2.1* and thereby electrically silence excitatory input into all Or83b-target glomeruli for the first 48 h after eclosion. Combining *Or83b-Gal4; UAS-Kir2.1* flies with a *Tub-Gal80^ts^* transgene expressing a temperature-sensitive repressor of GAL4, allowed conditional expression of Kir2.1 channels. When flies were kept at 29°C, the repressor was inactivated, but not at temperatures <21°C when the repressor blocks GAL4 function (McGuire et al., 2004). For the experiments, flies were grown at 18°C until eclosion to allow OSNs to develop normally and then shifted to 29°C silence OSNs for the first 48 h after eclosion (Chiang et al., 2009). After 2 d at 29°C, the 48- 60-h-old flies were exposed to EB for 4 d at RT (∼21°C) and their glomerular volumes were compared with those of two classes of control flies whose OSNs remained fully functional. First, flies having the transgenes Or83b-Gal4 as well as UAS Kir2.1; Tub-Gal80ts were separated into two groups and aged for 48 h either at RT or at 29°C. And second, *UAS-Kir2.1; Tub-Gal80^ts^* flies that lacked the GAL4 transgene were treated in the identical way as the experimental flies.

**Figure 5.**
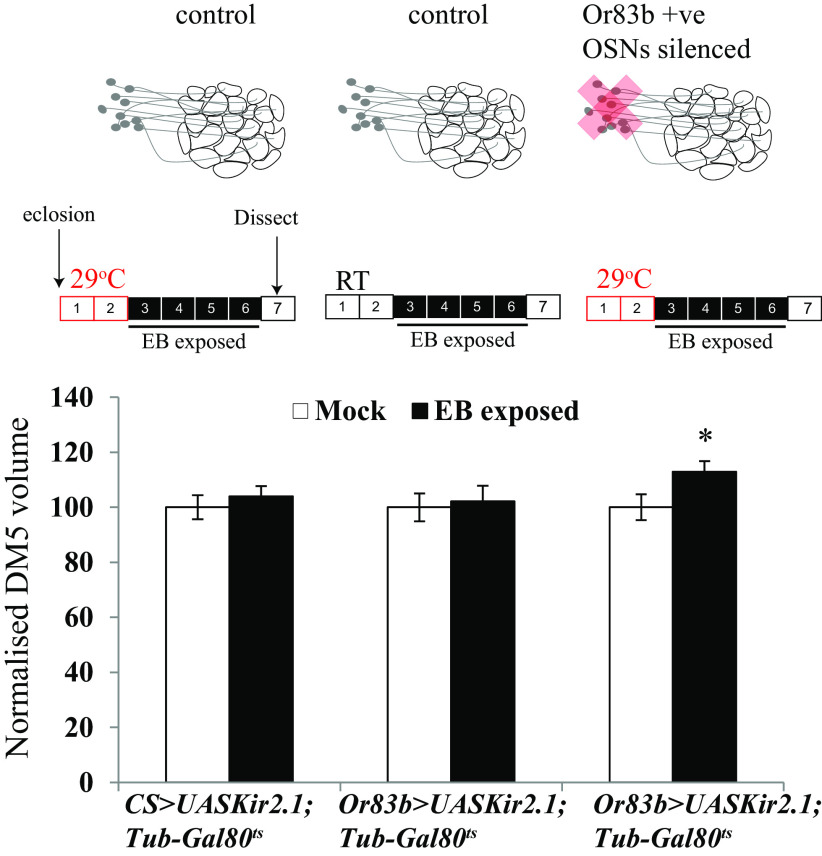
Silencing OSNs during the critical period extends it. Schematic shows *Or83b-Gal4*-positive sensory neurons, which are active during the CP in control flies and are silenced for 48 h after eclosion in *Or83b-Gal4* > *UAS-Kir2.1; TubGal80^ts^* flies. Schematics show days of odor exposure. The red boxes indicate that flies were aged at 29°C without odor exposure, and the black boxes indicate that flies were exposed to odor at room temperature. The numbers indicate days after eclosion. Normalized volumes of DM5 glomeruli of *Or83b-Gal4* > *UAS-Kir2.1; TubGal80^ts^* along with temperature and genotype controls. The white bar corresponds to mock exposure, whereas the black bar corresponds to EB exposure. Error bars indicate the mean ± SEM. *p ≤ 0.05, determined by Student's *t* test.

Consistent with previous experiments, control flies (CS > *UAS-Kir2.1; Tub-Gal80^ts^* and *Or83b-Gal4* > *UAS-Kir2.1; Tub-Gal80^ts^* maintained at 21°C) with functional sensory neurons exposed for 4 d to EB starting 48–60 h after eclosion showed no increase in the volume of DM5 glomerulus. In contrast, however, similarly aged and exposed flies whose OSNs had been silenced for the first 48 h showed a significant increase in DM5 volume (EB-exposed flies, 669.7 ± 23.69 µm^3^; mock-exposed flies, 538.5 ± 29.25 µm^3^; *p* < 0.043; Fig. 5, Table 3). Thus, silencing the activity of Or83b-positive OSNs extends the capacity for EB-evoked plasticity in the olfactory glomeruli beyond the normal CP.

This immediately raised the question of whether OSN silencing would have local effects on CP in silenced glomeruli or more general effects in the antennal lobe, an issue we addressed with additional experiments.

### Local control of critical period closure in the antennal lobe

In the *Drosophila* olfactory system, individual antennal lobe glomeruli differ not only in terms of the distinctive OSN and PN classes for each glomerulus, but also in biochemical and physiological properties of excitation and inhibition (Vosshall et al., 2000; Hong and Wilson, 2015; Seeholzer et al., 2018). Besides, some glomeruli mediate aversive responses while others mediate approach behaviors (Root et al., 2008; Semmelhack and Wang, 2009). To directly test whether the regulation of CPs for odorant-induced long-term plasticity could be determined at the level of individual glomeruli, we asked whether silencing the DM5 innervating *Or85a* class of OSN responding to EB would selectively extend the CP for EB-induced plasticity in the DM5 glomerulus without affecting closure of the CP for other adjacent EB-responsive glomeruli or for CO_2_-induced plasticity in the V glomerulus.

**Figure 6.**
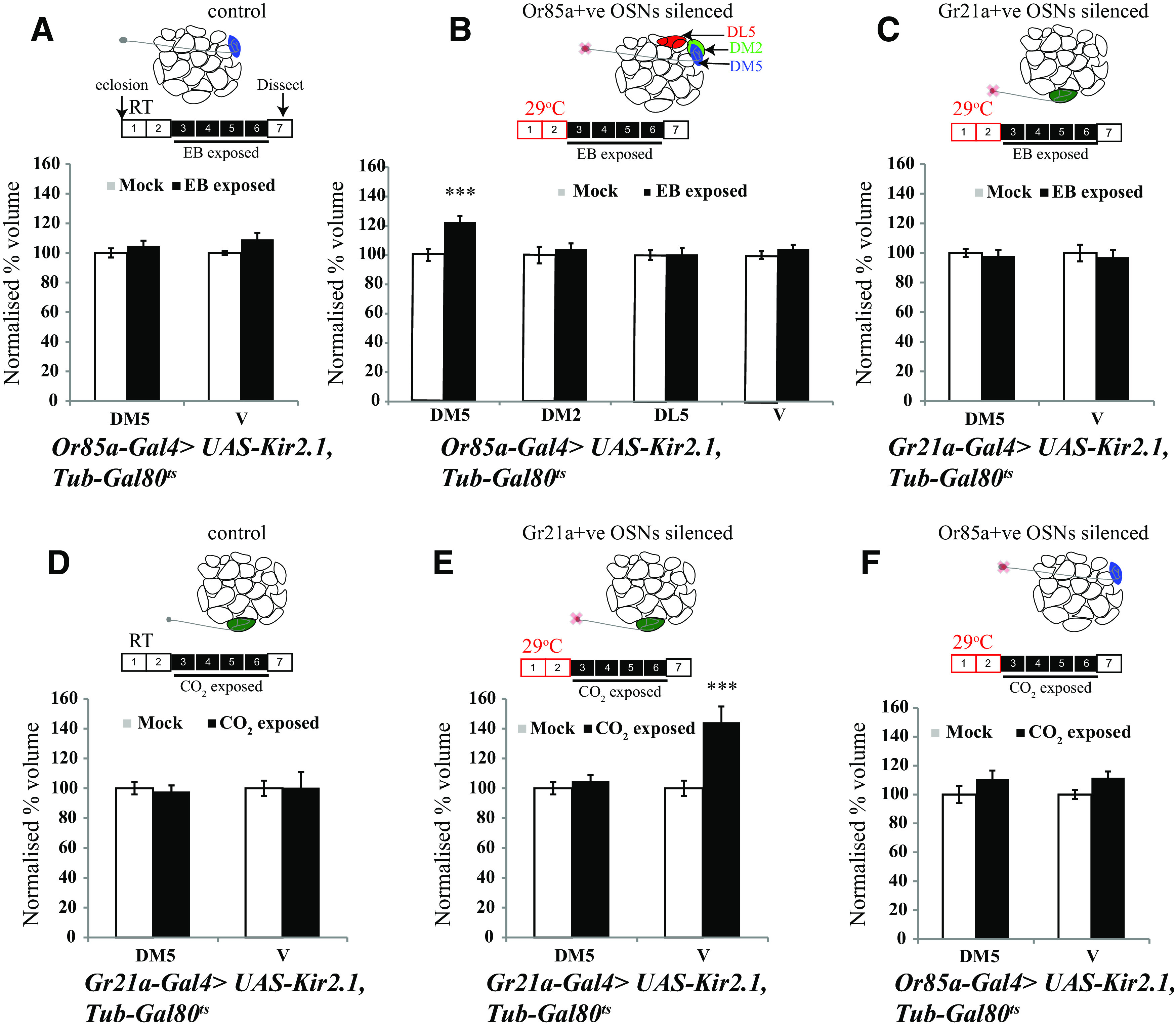
Local control of critical period closure. Schematics show days of odor exposure. The numbers indicate days after eclosion. The red boxes show the number of days flies were kept at 29°C, the empty boxes indicate flies were not exposed to odor, and the black boxes indicate that flies were exposed to odor at room temperature. ***A–F***, Normalized volumes of the indicated glomeruli of *Or85a-Gal4* > *UAS-Kir2.1; TubGal80^ts^* and *Gr21a-Gal4* > *UAS-Kir2.1; TubGal80^ts^* flies exposed to EB (***A–C***) and CO_2_ (***D–F***). Schematics of the antennal lobe above the respective bar graphs show the OSNs being silenced. The white bar corresponds to mock exposure, whereas the black bar corresponds to odor exposure. Error bars indicate the mean ± SEM. ***p ≤ 0.001, determined by Student's *t* test.

We expressed the inward-rectifying potassium channel Kir2.1 in either *Or85a-Gal4*- or *Gr21a-Gal4*-expressing OSNs to respectively silence either EB-responsive OSNs that innervate the DM5 glomerulus or CO_2_-responsive OSNs that innervate the V glomerulus. All flies, which also expressed GAL80^ts^, the temperature sensitive GAL4 inhibitor, were grown at 18°C until eclosion so that sensory neurons developed normally and were then shifted to 29°C posteclosion for 48 h, at which time point the CP for EB- and CO_2_-induced plasticity is closed in control flies (Figs. 5, 6).

Selective silencing of the OSNs that innervate the DM5 glomerulus or the V glomerulus for 48 h respectively led to odorant- and glomerulus-specific extension of CPs for EB- or CO_2_-induced structural plasticity. Thus, following 48 h of DM5 silencing (*Or85a-Gal4* > *UAS-Kir2.1; Tub-Gal80^ts^* maintained at 29°C), 4 d of EB exposure results in an increase in the DM5 glomerular volume (717.56 ± 29.48 µm^3^ in mock vs 880.71 ± 30.69 µm^3^ in EB-exposed, *p* < 0.001), but no detectable increase in volumes of flanking EB-responsive DM2 glomerulus and EB nonresponsive DL5 glomerulus compared with mock-exposed controls (Fig. 6). In addition, silencing of *Or85a* neurons did not extend the critical period for CO_2_-induced V glomerulus growth (2564 ± 82.62 µm^3^ in mock vs 2859 ± 115.55 µm^3^ in CO_2_ exposed, *p* = 0.239; Fig. 6*A–C*, Table 3). On similar lines, after 48 h silencing of Gr21a-positive OSNs, 4 d of exposure to CO_2_ results in an increase in the volume of the V glomerulus (2589 ± 132.75 µm^3^ in mock vs 3734.5 ± 274.65 µm^3^ in CO_2_ exposed, *p* < 0.001), but 4 d of EB exposure does not have any effect on the volume of DM5 (1157.78 ± 31.91 µm^3^ in mock vs 1133.25 ± 46.82 µm^3^ in EB exposed, *p* = 0.239; Fig. 6*D–F*).

**Figure 7.**
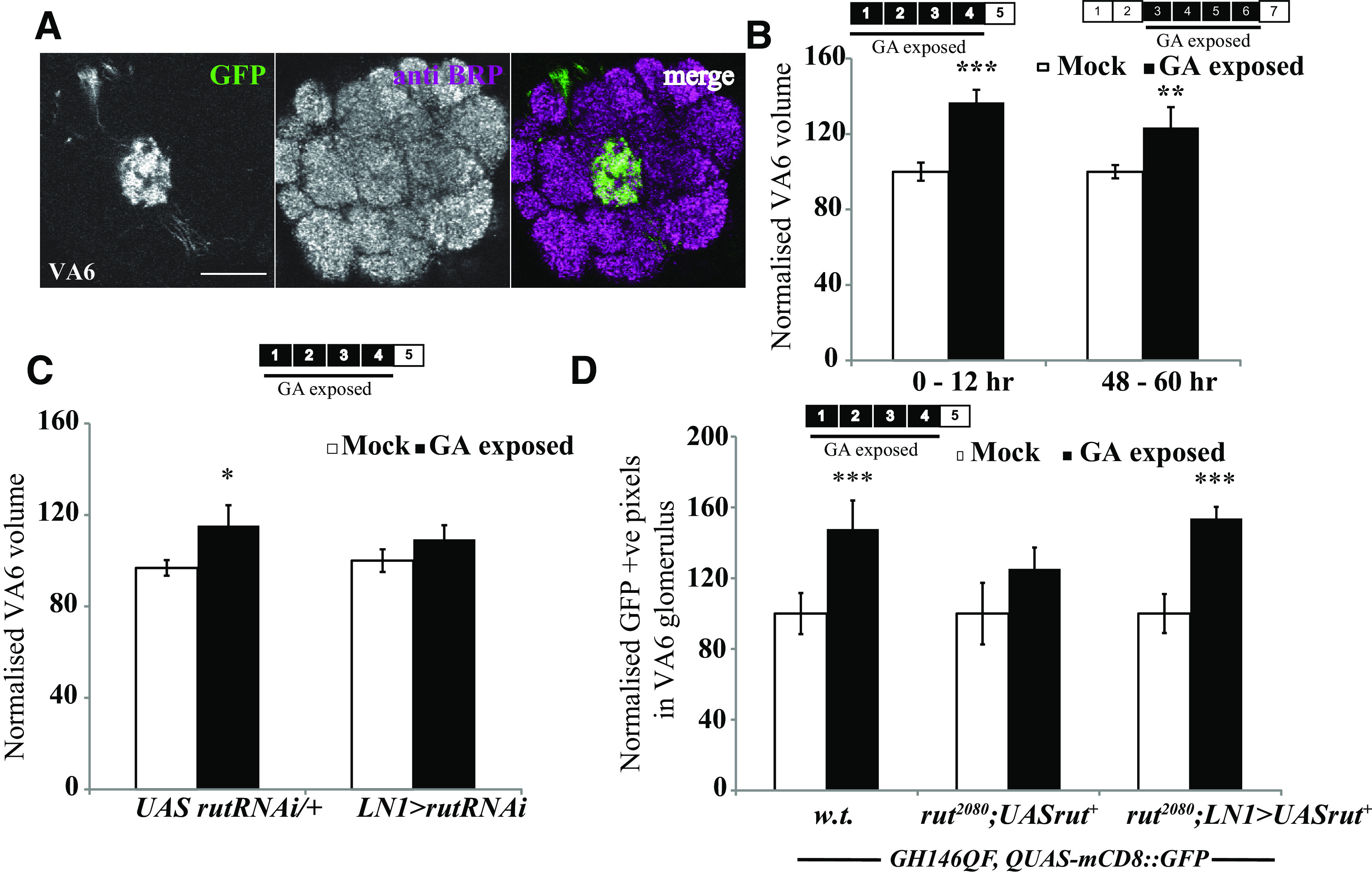
Adaptation induced by GA requires *rut* but does not show a critical period. ***A***, Image showing *Or82a-Gal4* > *UASmCD8::GFP*, which labels the GA-sensitive OSNs, innervating the VA6 glomerulus. ***B***, Schematics show days of odor exposure. The numbers indicate the number of days after eclosion. The black boxes indicate that flies were exposed to odor. Normalized volumes of VA6 glomeruli of *Or82a-Gal4* > *UAS mCD8::GFP* flies. ***C***, Normalized volume of VA6 glomerulus for *LN1-Gal4* > *UAS-rutRNAi* and *UAS-rutRNAi*/+ flies exposed to mock and GA for 4 d after eclosion. ***D***, GH146-positive PNs labeled with the Q system, *rut^2080^* mutants and flies having a wild-type *rut* expressed in *LN1-Gal4*-positive neurons in a *rut* mutant background. The white bar corresponds to mock exposure, whereas the black bar corresponds to GA exposure. Error bars indicate the mean ± SEM. *p ≤ 0.05, **p ≤ 0.01, ***p ≤ 0.001, determined by Student's*t* test.

Thus, critical period plasticity in the *Drosophila* antennal lobe can be regulated in a glomerulus-autonomous manner or at least a region-specific manner in the antennal lobe. We further tested whether this could explain the previously described absence of a CP for GA-induced plasticity (Fig. 7; Kidd et al., 2015).

### Long-term plasticity induced by the attractive odorant geranyl acetate involves the same cellular mechanisms as repulsive CO_2_ and EB but shows no critical period

Previous work has shown that 4 d of exposure of *Drosophila* to the attractive odorant GA induces growth of the GA-responsive VA6 glomerulus (Lieber et al., 2011). Unexpectedly, Kidd and Lieber (2016) showed GA-induced VA6 plasticity when exposure was initiated in 2- to 8-d-old flies, effectively arguing against the presence of a CP for long-term olfactory plasticity. This conundrum could be explained by postulating glomerulus-specific differences in CPs.

We first repeated previous experiments showing that the CP for GA remains open beyond 2 d. These experiments confirmed that flies exposed to GA for 4 d starting 48–60 h (1730 ± 61 µm^3^ in mock vs 2341 ± 188 µm^3^ in GA exposed, *p* < 0.014) or 0–12 h (1712 ± 82 µm^3^ in mock vs 2134 ± 110 µm^3^ in GA-exposed flies, *p* < 0.001) after eclosion showed an increased volume of VA6 glomerulus compared with mock-exposed controls (Fig. 7*B*). Thus, we independently confirm that the CP for GA-induced plasticity of the VA6 glomerulus remains open at a time when it is closed for EB and CO_2_.

We then asked whether the mechanism for plasticity induced by long-term odor exposure was different for GA, since it does not have the same critical period for odor-induced plasticity. Specifically, we asked whether adenylate cyclase function in inhibitory local interneurons (iLNs) was required for GA induced glomerular growth, as shown for EB- and CO_2_-induced structural plasticity.

We expressed the RNAi against *rut* in the LN1 subset of interneurons to knock down adenylate cyclase function to see whether this affected GA-induced VA6 glomerular growth. Consistent with *rut* function in iLNs being necessary (Fig. 4), we observed no significant GA-induced VA6 growth in *LN1-Gal4* > *UAS-rutRNAi* flies after 4 d GA exposure (Fig. 7*C*). We also found that *rut^2080^* mutants failed to display PN plasticity after 4 d of GA exposure (*rut^2080^; GH146-QF, QUAS-mCD8::GFP*), but expressing wild-type *rutabaga* in the LN1 neurons in whole-body *rut* mutants (*rut^2080^; LN1-Gal4/UAS-rut^+^; GH146-QF, QUAS-mCD8::GFP*) is sufficient to rescue GA-induced PN plasticity (Fig. 7*D*). Hence, plasticity induced by long-term exposure to the attractive odorant GA shares a key common cellular mechanism with that induced by the repulsive odorants EB or CO_2_ (Figs. 4, 7*C*,*D*; Das et al., 2011).

The lack of CP closure for GA-induced plasticity provides further proof of the ability for local regulation of CPs at the level of individual glomeruli.

## Discussion

### Activity-dependent regulation of critical period closure in the antennal lobe

To understand how the CPs for plasticity are determined and how these could potentially differ across glomeruli (Figs. 5, 6, 7), we tested whether neural mechanisms influenced closure of the CPs in olfactory glomeruli.

It is believed that younger animals have plasticity in their neural circuits to allow them to adapt to the specific environment they are in, but there are brakes on plasticity that actively prevent extensive neuronal remodeling in mature animals, thus allowing for stability of their neural connections (Takesian and Hensch, 2013). In mammalian systems, late-developing perineuronal nets, which protect neurons (Cabungcal et al., 2013), may provide physical mechanisms for restricting the plasticity observed in juvenile animals in favor of stability that may be adaptive in mature nervous systems (Carstens et al., 2016).

In mammalian visual systems, neural activity is important not only for proper wiring of neurons in the visual system during the CP, but also for the CP closure (Mower, 1991; Hensch, 2004). Thus, rearing animals in the dark slows down the progression of the CP and allows plasticity to be induced in older animals (Mower, 1991; Fagiolini et al., 1994; Desai et al., 2002). While the exact mechanism for this has yet to be elucidated, several arguments implicate the activity of inhibitory GABAergic interneurons in bidirectional control of CP closure (Benevento et al., 1992, 1995; Huang et al., 1999; Gianfranceschi et al., 2003).

Our work shows for the first time that electrical activity in underlying circuits also regulates critical period closure in *Drosophila*. In support, experimental silencing of OSNs, which should reduce activity in downstream inhibitory and excitatory neurons in the antennal lobe, causes the critical period for long-term olfactory plasticity to stay open for longer than in control animals (Figs. 5, 6). Thus, the phenomenon of activity-dependent closure of CPs appears to be conserved from *Drosophila* to mammals.

Given that activity is important in closing the CP, we studied the effect of specific OSN silencing, which would effectively deprive the animals of olfactory stimulus, on the CP. This allowed us to study the effect of perturbations in different classes of OSNs, responding to specific odorants, to determine how the CP is regulated in the *Drosophila* olfactory system.

### Local regulation of critical periods

The 48 h CP for CO_2_- and EB-induced plasticity in V and DM5 glomeruli, respectively, is not observed for GA-induced plasticity in VA6 glomerulus. The organization of these glomeruli is superficially similar, receiving processes from glomerular-specific OSNs, PNs, and largely common populations of LNs. What then is the underlying difference in CP regulation? And why might such flexibility exist in the brain?

Since V, VA6, and DM5 are within 20 μm of each other, this indicated somewhat local control of CP plasticity in the antennal lobe. Additional experiments pointed to the existence of glomerular autonomous mechanisms for CP closure. We showed that electrical silencing of OSNs, which exclusively target DM5, extends the CP for DM5 plasticity but not for the adjacent EB-responsive DM2 glomerulus (Fig. 6). Thus, glomerulus-specific features or mechanisms for critical period regulation could potentially account for the differences in CP previously described across reports on EB/CO_2_- or GA-evoked glomerular plasticity.

At a physiological level, this observation suggests either that patterns of odorant-evoked activity vary across glomeruli, or that different glomeruli show different sensitivities to extracellular factors, including neuromodulators and growth factors that mediate structural plasticity. Both of these predictions have experimental support. OSN classes vary widely in terms of the basal firing frequencies as well as in details of the intensity and duration of their responses to odorants (Hallem et al., 2004; Hallem and Carlson, 2006; Mathew et al., 2013; Seki et al., 2017). In addition, the sensitivity of different PNs and OSNs to inhibition, excitation, and modulation have been shown to vary across glomeruli. Distribution of GABA receptors and GABAergic regulation varies widely across individual olfactory glomeruli in the *Drosophila* antennal lobe (Root et al., 2008; Hong and Wilson, 2015; Seeholzer et al., 2018) and the projection of various neuromodulatory interneurons can show considerable glomerular specificity (Singh et al., 2013; Kayser et al., 2014). Thus, though superficially similar in circuit design, glomerular input, glomerular output, and plasticity properties can show wide variation, which could explain the difference in CP control of GA- and CO_2_/EB-induced plasticity.

### Mechanisms of neural plasticity are similar even when critical periods differ

Additional experiments tested whether crucial mechanisms for CO_2_ and EB LTH were also required for GA-induced plasticity in the VA6 glomerulus, since they induce similar increases in volumes of the odorant-selective glomeruli after prolonged odorant exposure, as well as increased arborization of PN processes in the odorant-selective glomeruli (Figs. 1, 3, 4, 7). Also that *rutabaga* function was required in LN1 neurons for the elaboration of PN processes induced by both EB and GA (Figs. 4, 7). These results indicate that mechanisms of habituation are similar, even when there are differences in odor valence or regulation of a critical period for plasticity.

Together, these observations are consistent with, but also extend, previous studies that have shown the presence of dendritic plasticity in the mammalian visual system in response to small lesions, at a time when the CP for conventional ocular dominance plasticity observed has closed (Keck et al., 2008; Hübener and Bonhoeffer, 2014). While observations in mammals show that critical periods can be regulated differently across large- and small-scale plasticity processes, our observations in the fly olfactory system show that a particular sensory channel can retain plasticity after the CP has closed in an immediately adjacent analogous channel. Given the experimental accessibility and relative simplicity of the *Drosophila* olfactory circuit, the antennal lobe may prove to be a valuable model system to study CP regulation with cellular resolution *in vivo*.

Several previous studies have >established that long-termbehavioral habituation to EB and CO_2_ occurs together with increased volume of odorant-responsive glomeruli, DM5 and V glomeruli, whose activation is required for behavioral aversion to respective odorants (Suh et al., 2004; Sachse et al., 2007; Semmelhack and Wang, 2009; Das et al., 2011; Sudhakaran et al., 2012). All known genetic requirements for olfactory LTH, including the need for cAMP signaling in inhibitory LNs, are also required for associated glomerular growth and the temporal features of LTH closely match those for of DM5 and V glomeruli as volume increases (Das et al., 2011; McCann et al., 2011; Sudhakaran et al., 2012). Moreover, reduced odorant-evoked responses in EB- or CO_2_-responsive projection neuron dendrites are associated with long-term olfactory habituation (Sachse et al., 2007; Das et al., 2011; McCann et al., 2011; Sudhakaran et al., 2014). Together, these data strongly support a causal connection between anatomic and physiological changes in DM5 or V glomeruli and behavioral LTH (Sachse et al., 2007; Glanzman, 2009; Das et al., 2011; Sudhakaran et al., 2012, 2014). While other glomeruli, such as the recently studied VM7 glomerulus, may also show alternative and intriguing forms of structural plasticity in response to odorant exposure, additional experiments are required to establish the underlying mechanisms as well as their relevance, if any, to behavioral habituation (Golovin et al., 2019; Fig. 2, Table 3)

Our finding that plasticity driven by adenylate cyclase activity in inhibitory local interneurons is required for long-term plasticity induced by both EB and GA, which are aversive and attractive odorants, respectively, suggests that identical signaling pathways, leading to increased inhibition, onto odorant-responsive PNs following long-term odorant exposure can account for the habituation of both aversive and attractive responses mediated by respective projection neurons. This is consistent with observations in larvae (Larkin et al., 2010).

While additional experiments on multiple odorants, both aversive and attractive, will be required to test this hypothesis, we speculate that differences in the regulation of CPs for GA- and EB/CO_2_-induced plasticity point to an evolutionarily useful adaptation that allows flies to retain the ability to habituate to innately attractive but useless odorants encountered in the lifetime of the animal.
