# Supplementary material for: Glomerulus-Selective Regulation of a Critical Period for Interneuron Plasticity in the Drosophila Antennal Lobe
Source: J Neurosci. 2020 Jul 15;40(29):5549–60. doi: 10.1523/JNEUROSCI.2192-19.2020 (PMC7363474; doi:10.1523/JNEUROSCI.2192-19.2020)
Supplement: Table 3-1: — Glomerular volume values for indicated genotypes and experimental conditions. Volumes of glomeruli (in μm3) of the indicated genotype after 4 d of odor exposure starting at 0–12, 24–36, and 48–60 h after eclosion. Download Table 3-1, DOC file. [file ns-JN-RM-2192-19-s01.doc]

**Extended Table 3-1:** Glomerular volume values for indicated genotypes and experimental conditions.

| Genotype | Temperature | Exposure started at | Exposed to | Glomeruli measured | Volume in micron^3 | | p value |
| --- | --- | --- | --- | --- | --- | --- | --- |
|  |  |  |  |  | Mock exposed (n) | Odor exposed (n) |  |
| *CS* | RT | 0-12 hr | EB | DM5 | 933.48 ± 29.25 (25) | 1116.93 ± 34.25 (27) | 2.82E-06 |
| *CS* | RT | 0-12 hr | EB | V | 3372.57 ± 187.11 (14) | 3471.31 ± 241.1 (13) | 0.7035 |
| *CS* | RT | 0-12 hr | EB | DM2 | 1947.29 ± 59.81 (17) | 2084.46 ± 47.62 (20) | 0.0476 |
| *CS* | RT | 0-12 hr | EB | VM7 | 736.28 ± 35.52 (7) | 728.25 ± 8.94 (8) | 0.737 |
| *CS* | RT | 24-36hr | EB | DM5 | 809 ± 21.75 (10) | 1016.86 ± 34.32 (7) | 0.00037 |
| *CS* | RT | 24-36hr | EB | V | 2242.83 ± 62.5 (6) | 2240 ± 67.7 (7) | 0.9760 |
| *CS* | RT | 48-60hr | EB | DM5 | 725 ± 10.52 (19) | 710.68 ± 11.73 (22) | 0.3690 |
| *CS* | RT | 48-60hr | EB | V | 1799.44 ± 23.08 (9) | 1892.56 ± 13.43 (9) | 0.0344 |
| *CS* | RT | 0-12 hr | CO_2_ | V | 2205 ± 63.82 (12) | 3105.5 ± 159.26 (14) | 6.54E-05 |
| *CS* | RT | 0-12 hr | CO_2_ | DM5 | 1078.29 ± 24.27 (7) | 1069 ± 29.79 (10) | 0.8111 |
| *CS* | RT | 24-36hr | CO_2_ | V | 2301 ± 37.85 (8) | 2715 ± 147.39 (6) | 0.03242 |
| *CS* | RT | 24-36hr | CO_2_ | DM5 | 747.83 ± 54.47 (7) | 705.67 ± 30.88 (7) | 0.5118 |
| *CS* | RT | 48-60hr | CO_2_ | V | 2260.33 ± 55.68 (12) | 2332.08 ± 40.4 (12) | 0.3094 |
| *CS* | RT | 48-60hr | CO_2_ | DM5 | 1018.4 ± 41.2 (10) | 1066.8 ± 37.4 (8) | 0.3539 |
| *CS>Kir2.1;tubGal80^ts^* | 29C | 48-60hr | EB | DM5 | 486.09 ± 21.27 (11) | 505.43 ± 18.14 (14) | 0.4966 |
| *CS>Kir2.1;tubGal80^ts^* | 29C | 48-60hr | EB | V | 2070.41 ± 96.29 (12) | 2139.67 ± 73.77 (9) | 0.5748 |
| *Or83b>Kir2.1;tubGal80^ts^* | RT | 48-60hr | EB | DM5 | 623 ± 31.49 (7) | 636.71 ± 35.18 (7) | 0.7765 |
| *Or83b>Kir2.1;tubGal80ts* | RT | 48-60hr | EB | V | 2156.25 ± 79.27 (6) | 2177.5 ± 81.05 (8) | 0.8557 |
| *Or83b>Kir2.1;tubGal80^ts^* | 29C | 48-60hr | EB | DM5 | 538.5 ± 29.25 (10) | 669.7 ± 23.69 (19) | 0.0428 |
| *Or83b>Kir2.1;tubGal80^ts^* | 29C | 48-60hr | EB | V | 2148.8 ± 67.7 (5) | 2121.88 ± 21.27 (8) | 0.8345 |
| *Or85a>Kir2.1;tubGal80^ts^* | RT | 48-60hr | EB | DM5 | 1172.8 ± 36.08 (10) | 1228.3 ± 49.58 (9) | 0.3784 |
| *Or85a>Kir2.1;tubGal80^ts^* | RT | 48-60hr | EB | V | 3740.67 ± 53.42 (9) | 4156.13 ± 139.17 (8) | 0.0789 |
| *Or85a>Kir2.1;tubGal80^ts^* | 29C | 48-60hr | EB | DM5 | 717.56 ± 29.48 (18) | 880.71 ± 30.69 (21) | 0.0003 |
| *Or85a>Kir2.1;tubGal80^ts^* | 29C | 48-60hr | EB | DM2 | 1629.75 ± 91.89 (8) | 1695.75 ± 64.57 (12) | 0.5664 |
| *Or85a>Kir2.1;tubGal80^ts^* | 29C | 48-60hr | EB | DL5 | 1411.5 ± 47.03 (10) | 1418.7 ± 60.34 (10) | 0.9261 |
| *Or85a>Kir2.1;tubGal80^ts^* | 29C | 48-60hr | EB | V | 2514.14 ± 67.96 (14) | 2619.8 ± 68.68 (15) | 0.2838 |
| *Or85a>Kir2.1;tubGal80^ts^* | 29C | 48-60hr | CO_2_ | V | 2564 ± 82.62 (8) | 2859 ± 115.55 (8) | 0.2389 |
| *Or85a>Kir2.1;tubGal80^ts^* | 29C | 48-60hr | CO_2_ | DM5 | 833.43 ± 49.69 (7) | 950.5 ± 49.81 (6) | 0.0588 |
| *Gr21a>Kir2.1;tubGal80^ts^* | RT | 48-60hr | CO_2_ | V | 2593.47 ± 84.87 (15) | 2602.6 ± 209.31 (10) | 0.9684 |
| *Gr21a>Kir2.1;tubGal80^ts^* | RT | 48-60hr | CO_2_ | DM5 | 984.63 ± 36.97 (8) | 962.71 ± 91.15 (7) | 0.8293 |
| *Gr21a>Kir2.1;tubGal80^ts^* | 29C | 48-60hr | CO_2_ | V | 2589 ± 132.75 (14) | 3734.5 ± 274.65 (16) | 0.0011 |
| *Gr21a>Kir2.1;tubGal80^ts^* | 29C | 48-60hr | CO_2_ | DM5 | 830.25 ± 34.14 (12) | 954.27 ± 64.3 (11) | 0.4456 |
| *Gr21a>Kir2.1;tubGal80^ts^* | 29C | 48-60hr | EB | V | 2820.83 ± 129.63 (6) | 2739.83 ± 97.68 (6) | 0.0588 |
| *Gr21a>Kir2.1;tubGal80^ts^* | 29C | 48-60hr | EB | DM5 | 1157.78 ± 31.91 (9) | 1133.25 ± 46.82 (12) | 0.2389 |
